# Forest Fire Detection Using New Routing Protocol

**DOI:** 10.3390/s22207745

**Published:** 2022-10-12

**Authors:** Fahad Taha AL-Dhief, Ravie Chandren Muniyandi, Naseer Sabri, Mosab Hamdan, Nurul Mu’azzah Abdul Latiff, Musatafa Abbas Abbood Albadr, Mutaz Hamed Hussien Khairi, Muzaffar Hamzah, Suleman Khan

**Affiliations:** 1 Faculty of Engineering, School of Electrical Engineering, Universiti Teknologi Malaysia (UTM), Johor Bahru 81310, Malaysia; 2 Center for Software Technology and Management, University Kebangsaan Malaysia (UKM Bangi), Bangi 43600, Malaysia; 3 College of Technical Engineering, Al-Farahidi University, Baghdad 14132, Iraq; 4 Faculty of Computing and Informatics, Universiti Malaysia Sabah, Kota Kinabalu 88400, Malaysia; 5 CAIT, Faculty of Information Science and Technology, Universiti Kebangsaan Malaysia, Bangi 43600, Malaysia; 6 School of Psychology and Computer Science, University of Central Lancashire, Preston PR1 2HE, UK

**Keywords:** node route busyness, forest fire detection, location-aided routing, lar-based reliable routing protocol

## Abstract

The Mobile Ad-Hoc Network (MANET) has received significant interest from researchers for several applications. In spite of developing and proposing numerous routing protocols for MANET, there are still routing protocols that are too inefficient in terms of sending data and energy consumption, which limits the lifetime of the network for forest fire monitoring. Therefore, this paper presents the development of a Location Aided Routing (LAR) protocol in forest fire detection. The new routing protocol is named the LAR-Based Reliable Routing Protocol (LARRR), which is used to detect a forest fire based on three criteria: the route length between nodes, the temperature sensing, and the number of packets within node buffers (i.e., route busyness). The performance of the LARRR protocol is evaluated by using widely known evaluation measurements, which are the Packet Delivery Ratio (PDR), Energy Consumption (EC), End-to-End Delay (E2E Delay), and Routing Overhead (RO). The simulation results show that the proposed LARRR protocol achieves 70% PDR, 403 joules of EC, 2.733 s of E2E delay, and 43.04 RO. In addition, the performance of the proposed LARRR protocol outperforms its competitors and is able to detect forest fires efficiently.

## 1. Introduction

MANET is considered an infrastructure-less network that involves many mobile networked devices that are referred to as nodes. The MANET’s nodes are capable of moving in different directions and speeds because these nodes are organized differently [1]. Each node has its transmission range that makes it able to connect with its neighbor nodes. However, the source node may be located at a distance farther than the destination node. Consequently, nodes within the MANET cooperate to transmit a packet and deliver it to the destination node [2]. This is because each node can only intercommunicate with other nodes that are situated inside its transmission radius.

**Figure 1:**
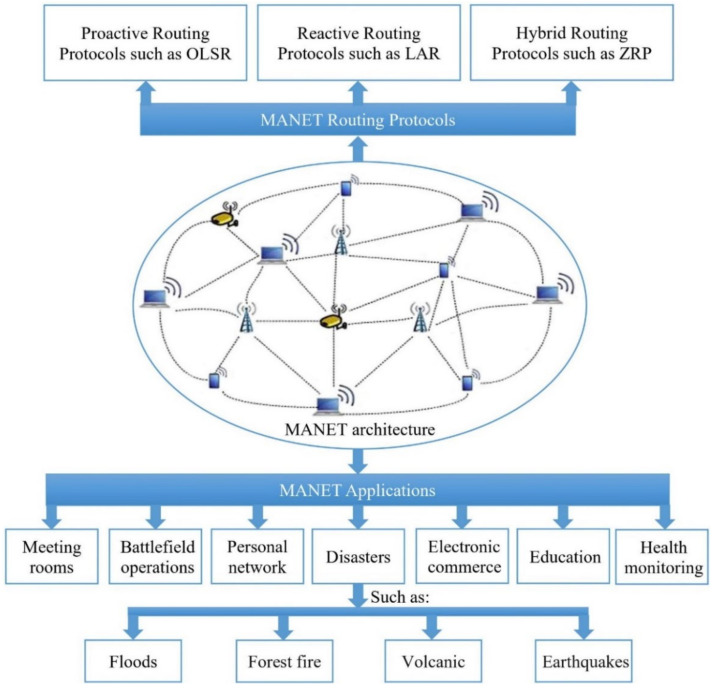
The MANET routing protocols and its applications.

Routing is a technique used in the MANET’s network to share node information with each other [3]. Due to the limited transmission range of MANET’s nodes, wireless radios routing is generally performed by multi-hops, where the information is routed through intermediate mobile nodes [4]. In MANET, the routing protocols forward the packets by intermediate nodes in order to send packets and deliver them to the target node [5]. Generally, MANET’s protocols are classified into three major types, namely Reactive (e.g., Location-Aided Routing (LAR)) [6], Proactive (e.g., Optimized Link State Routing (OLSR)), and Hybrid (e.g., Zone Routing Protocol (ZRP)) [7]. Many different routing protocols of MANET have been developed and proposed for the purpose of enhancing the network’s performance with respect to reducing packet loss, to reducing the average time delay for transmitting packets, or to prolonging the lifetime of the MANET’s network [8]. MANET has many applications in different domains of our daily life such as meeting rooms, personal area networks, commercial applications, battlefield communication, education, and healthcare [9,10,11]. Furthermore, MANET has a significant role in many applications of emergency and disasters such as flood detection [12], forest fire detection [13], volcanic eruptions [14], and earthquakes [15]. Figure 1 shows the MANET architecture, its applications, and the main types of routing protocols.

Forest fires are considered one of the largest risks threatening human life and forest resources. It is known that forest fires spread over significant distances and continue for weeks or several months, which bring many environmental risks and often cause air pollution to neighboring countries [16]. However, the forests are generally located in remote and unmanaged areas. In addition, the forests are mostly filled with dry wood, trees, and leaves that are considered fuel sources for the fire. Forest fires may be ignited due to climatic changes or human activities [17]. Moreover, the forest fire is observed only when it has already spread through a large area, making the process of stopping these fires very difficult [18]. Thus, these fires result in devastating losses and large damages to both the environment and atmosphere. Additionally, forest fires lead to long-term negative effects, for instance, forest fires have an effect on global warming, weather patterns, and rare species extinction of fauna and flora. Consequently, forest monitoring is imperative in order to avoid forest fire consequences [19]. Nevertheless, the network of forest fire detection consumes a significant amount of power because this network performs continuous long-time monitoring of the forest. The mobile sensor network is used for forest fire detection due to its many advantages: the network’s sensors can sense fire heat, this network can be installed at any time and place, it can present access to information of the sensors’ position, it is a self-configuring network, the sensor nodes may work as routers, it is cheaper than wired sensor networks, it exhibits scalability and flexibility, and this mobile sensor network is considered robust because its administration is decentralized [20,21].

However, the routing protocols in the area of forest fire networks are still too inefficient with respect to sending data and energy consumption, which limits the lifetime of the network for forest monitoring. Therefore, this paper presents a developed routing protocol called a LAR-Based Reliable Routing Protocol (LARRR). The presented LARRR protocol considers the route length between nodes, the temperature sensing, and the node’s buffer for improving the network performance of forest fire monitoring. Additionally, the performance of LARRR protocols is evaluated by using different evaluation measurements.

This paper is organized as follows: Section 2 presents the related works of forest fire techniques. Section 3 presents the conventional method used in the detection of forest fires. Section 4 provides a review of the LAR protocol. Section 5 presents the proposed LARRR protocol. Section 6 presents the simulation setup of the LARRR protocol. Section 7 provides the evaluation measurements. Section 8 gives and discusses the results of the LARRR protocol. Finally, Section 9 presents the conclusion and future works.

## 2. Related Work

Recently, many developers and researchers have had a significant interest in managing and monitoring forest fires to avoid disasters that result in much destructive damage that is perhaps irreparable. They have used several methods and different routing protocols such as those in [22,23,24,25], where their methods aimed to improve the network performance of forest fire detection. Here, we review some recent methods used in the detection of wildfires.

A detection system of forest fires relying on Wireless Sensor Networks (WSNs) was proposed in [26]. There are three main stages used in this system: the sensing stage, routing stage, and communication stage. In the first stage, two sensors called smoke and fire are used for sensing the physical change (i.e., methane, propane, and combustible gases) in the forest environment as well as for early detection of a forest fire. When these two sensors sense or detect something, they will transmit data to the master node through utilizing Radio Frequencies (RFs); this process is considered the second stage. In the third stage, the master node will analyze data received from all nodes, where if a node senses or detects a fire, the master node will communicate directly with the base station over a Global System for Mobile Communication (GSM) modem. GSM is able to send and receive messages and can also be linked to a computer. Subsequently, the base station will be alerted where the user can reach it. Furthermore, OMNeT++ simulation software was used in this system for forest fire monitoring. However, the performance of this system was not assessed by PDR or EC.

Another new approach was presented for forest fire detection by using different routing protocols of WSNs [27]. The routing protocols used in this method are EAQHSeN, HDMRP, and Multilevel. This method aims to compare these protocols in four scenarios of forest fire detection as well as to evaluate their performance with respect to the network response time and the network lifetime. These routing protocols were performed by using the Castalia WSNs simulator, and the results showed that their performance was very similar with respect to the energy consumption with a small advantage for EAQHSeN and HDMRP protocols. Furthermore, the performance of EAQHSeN and HDMRP protocols was faster than the Multilevel protocol with respect to the network response time. However, this method did not conclude which of these protocols achieved the best results in the detection of the forest fire. Additionally, there was no improvement for any type of these routing protocols.

An energy-efficient technique was proposed for forest fire detection by using a WSN [28]. In this method, the authors used a scheme for three multi-sink sensor nodes. They aimed to amortize the network deployment cost with no adverse effects on the network performance. A node sends its sensed data to these three sink nodes by utilizing a data forwarding scheme. In addition, this method transmits the data by using three different modes: time-driven, event-driven, and hybrid mode. In the first mode, all nodes can sense relative humidity, temperature, and solar radiation. Subsequently, these data will be sent in a round-robin fashion to three sink nodes. In the second mode, a node uses a random transmission mode and responds only when the temperature is recorded above the threshold of 50 °C. Meanwhile, the third mode is a mixture of event-driven and time-driven modes. In other words, it works similar to the first mode in terms of data transmission in the form of a round-robin, and it works similar to the second mode when the temperature crosses the determined threshold. In addition, the third mode can transmit the data of wind direction, where the fire spread direction can be expected in the environment. According to the results, the hybrid mode achieved the highest lifetime of the network due to it using the best features of the other two modes. However, this method does not take into account the sleep mode of nodes, where a node can consume more energy when receiving and sending data.

A system of forest fire detection was presented by using a hardware design based on a Data Aggregation Routing Protocol (DARP) [29]. This design aims to achieve an energy-efficient WSN. In addition, there are four stages presented in this method, where the first stage is referred to as the user node that broadcasts the temperature data, the second scenario is the coordinator node, where this node will receive the temperature information, stores, and sends this information. Subsequently, in the third stage, the user node will store the temperature data in the cache for the purpose of speeding up the recall of temperature data. In the fourth stage, the destination node will respond to the temperature information via gradient routing. In addition, the results of this method showed that a 16 m distance is the best routing with the most energy efficiency. However, this method was implemented in a limited environment with four nodes only. Moreover, the energy of sensor nodes was not evaluated in terms of EC.

Another research study was proposed in [30], where the authors presented a cost-effective system for detecting wildfires early by employing narrow-beam Far-Infrared (FIR) sensors. This system aims to protect localized zones, for example, villages, camping places, or remote houses in the forest. The FIR sensor nodes are able to detect strong heat with a range of up to 50 m. Furthermore, these sensors are linked to STM32 L432KC nodes, which perform in a master–slave topology. The Slave nodes are used to read and pre-process temperature data by flame sensors and present these data to the master node. In addition, the master node will receive the temperature data, and because the master node is connected to the internet, it is responsible for forest monitoring and releasing the alarm in real-time. This system is applied with automatic countermeasures such as sprinklers for protecting small areas of interest exposed to forest fires. These sprinklers are activated with latching valves, which can maintain their status even if the power is lost.

In [31], the authors presented a new design based on WSNs for wildfire detection. This design aims to specify the scale and intensity of the forest fire by using the Fire Weather Index (FWI). In addition, a data communication solution is proposed in the simulation environment to create dynamic routing paths. There are different parameters (e.g., the weather index, the weight, the energy, and the security) that are used to develop these dynamic paths. In this system, the client application is presented and connected to the web services to update the received information from the WSN, where if a fire occurs in the forest, the client will specify the fire zone, temperature, index date, and wind speed. These parameters are used to calculate the FWI and are then sent to the base station. Furthermore, the Microsoft Framework was used to implement the simulation experiments. The results of this method showed the efficiency of detecting fires with fewer overhead data packets and less EC.

## 3. Conventional PCE-OLSR Method

Power Consumption Efficient-Optimized Link State Routing (PCE-OLSR) is a method proposed for forest fire surveillance by using mobile sensor networks [32]. The PCE-OLSR method is based on an OLSR protocol and it aims to increase the network’s lifetime by consuming node energy in an efficient manner. The technique of PCE-OLSR uses those nodes that are located in the fire area for the purpose of completely utilizing their energy before they burn and die by the fire. In this case, the other nodes that are located in safe areas do not use their energy. Therefore, the energy of those nodes may be conserved, continue working for as long as possible, and thus increase the network lifetime. Each node of the PCEOLSR has a threshold for temperature and it is set at 60 °C, where a node detects fire when its temperature reaches 60 °C. In other words, when a node’s temperature reaches this threshold, all its neighbors will change their routing to this node to exploit its energy before it is destroyed and dies by the fire.

**Figure 2:**
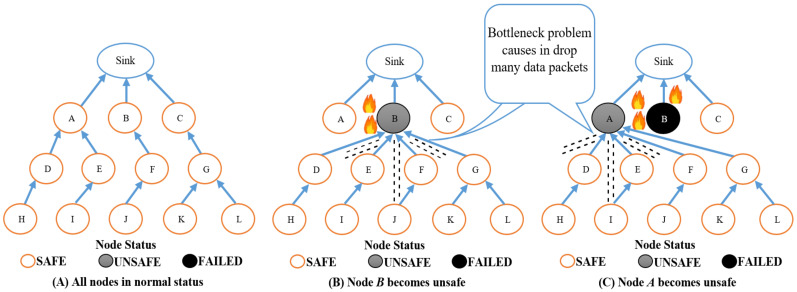
The PCE-OLSR diagram.

However, such a routing process can cause a bottleneck problem, as depicted in Figure 2. The temperature of the *B* node reaches 60 °C, as shown in Figure 2B. In this case, all neighbors of the *B* node (i.e., *D*, *E*, *F*, and *G* nodes) change their parent to the *B* node for the purpose of totally utilizing the energy of the *B* node. In Figure 2C, the temperature of node *B* reaches 130 °C and it is burned. In addition, the temperature of the *A* node reaches 60 °C, and therefore, *D*, *E*, *F*, and *G* nodes change their routing to *A* as well for the purpose of exploiting the *A* node’s energy. However, the bottleneck problem leads to accumulation of many data packets in the node’s buffer located in the fire area, wherever this node will be destroyed before transmitting all information. Consequently, many data packets will be dropped and lost in the network.

## 4. Concept of Location-Aided Routing Protocol (LAR)

**Figure 3:**
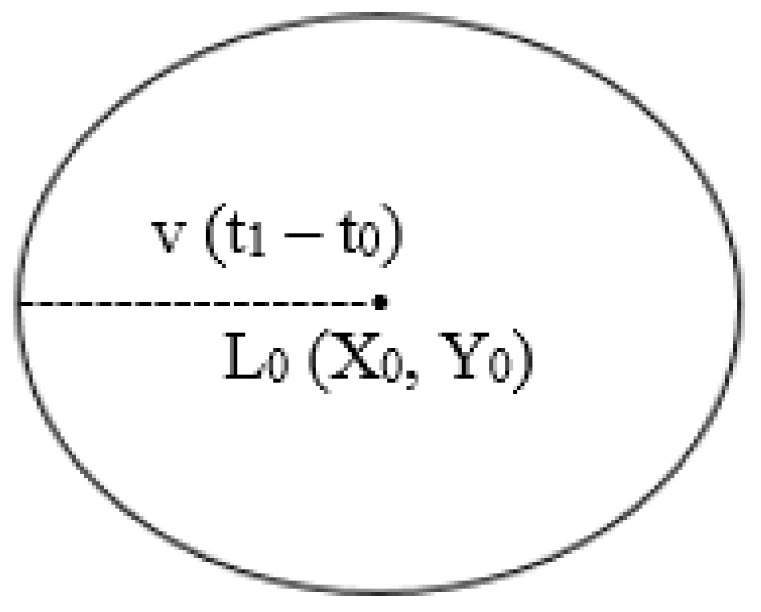
The expected zone.

Location-Aided Routing (LAR) is considered the first protocol that adopts the location information of mobile sensor nodes [33]. The routing process in the LAR protocol consists of two main nodes, which are the source node *S* and the destination node *D*. Generally, the *S* node in the LAR protocol has the location information of the *D* node by using Global Positioning System (GPS) technology, where the GPS technology presents the possibility of mobile sensor nodes knowing and sharing their physical location [34]. The region that has the *D* node is called the expected zone. Therefore, the *S* node shall take into account the predicted place of the *D* node at the location L_0_ (X_0_, Y_0_), at the time t_0_, and the average speed of the *D* node is V [35]. Consequently, the *S* node can suppose that the expected zone is a circular area of radius V (t_1_ − t_0_), and it is located at L_0_. Figure 3 illustrates the circular region of the expected zone.

**Figure 4:**
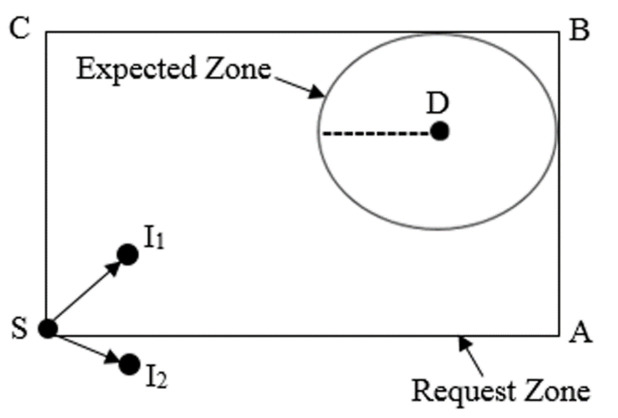
The request zone.

When the *S* node needs to transmit a packet to the *D* node, the *S* node must determine a route to the *D* node. In this case, the algorithm of the LAR protocol uses flooding by sending the route discovery process. Subsequently, the *S* node defines another region called the request zone, where the *D* node should be located inside the request zone. Figure 4 shows the request zone where its corners are C, B, S, and A. The *S* node can specify 4 corners of the request zone. Furthermore, when the *S* node needs a route to this zone, it will send a Route Request Message (RREQM) that includes the coordinates of the request zone.

**Figure 5:**
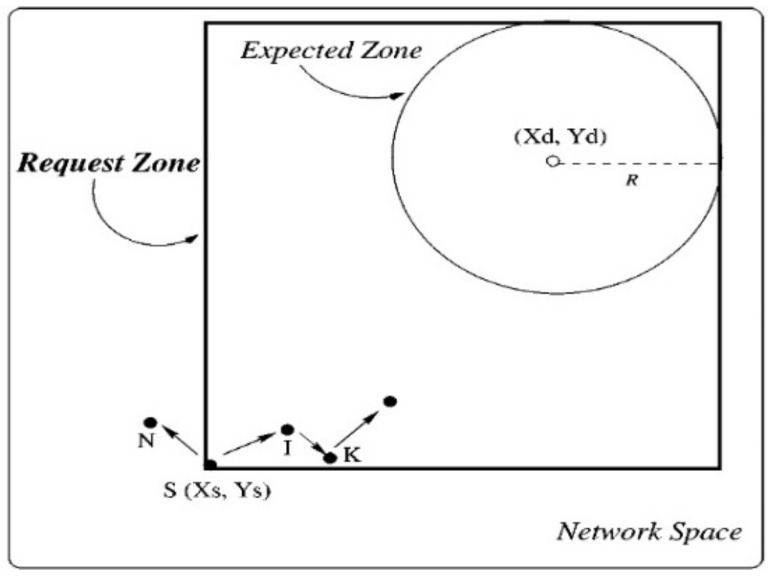
The expected and request zones.

When the *S* node sends the RREQM in the route discovery process, other nodes receive this route request only if they belong to the rectangle shape (i.e., the request zone). On the other hand, nodes that are located outside of the request zone will discard the RREQM. As shown in Figure 5, the RREQM is generated to form a route from the *S* node to the *D* node. This route request will be forwarded through *K* and *I* nodes because these nodes are located inside the request zone, while, when the *N* node obtains the route request, it will discard this RREQM because the *N* node is located outside the request zone.

Once the *D* node obtains the RREQM, it responds by transmitting the Route Reply Message (RREPM) to the *S* node. The RREPM will follow the same path that is obtained from the *S* node. In other words, the RREPM follows the reversed path of the RREQM. Continuing this process, the LAR protocol is considered to be energy-efficient with respect to the network lifetime as compared with conventional flooding algorithms, where it can decrease the cost of nodes routing in the network.

## 5. Methodology of LAR-Based Reliable Routing Protocol (LARRR)

Several protocols have been presented based on mobile sensor networks with the purpose of improving the network performance. The routing protocols differ in the manner of finding routes. The LAR protocol works efficiently with respect to consuming energy, where the process of discovering routes is initiated when there is a need for sending a packet. The routes that need to be created are performed by transmitting control packets over the network. As we mentioned previously, the LAR protocol defines and determines the request zone, where the nodes within this zone are authorized only to forward the control packets. Hence, the routing overhead will be reduced. According to the advantages of the LAR protocol, we select this protocol to be a base for our work. The major goal of this work is to develop the LAR protocol and make it perform efficiently and reliably in forest fire detection. The new routing protocol is named the LAR-Based Reliable Routing Protocol (LARRR). The LARRR protocol is adapted to route the packets between the mobile sensor networks for forest fire monitoring. Furthermore, every node of the LARRR can sense the temperature continuously. More information about finding the best routes is explained in the next subsection.

### Finding the Route

**Figure 6:**
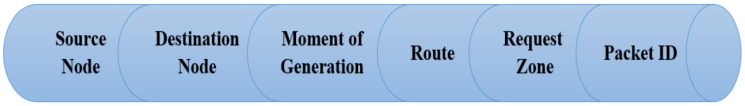
The contents of RREQM.

The route discovery process for building routes is initiated once the node generates a data packet and needs to send. In this case, the node should determine the request zone with respect to the place information obtainable within its location table. Subsequently, the node will be able to send the RREQM to other nodes within the network. In the proposed LARRR protocol, the RREQM will include the information illustrated in Figure 6.

**Figure 7:**
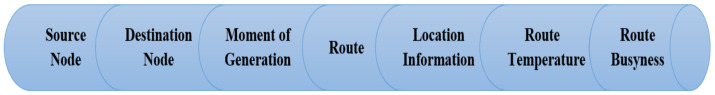
The contents of RREPM.

The RREQM is transmitted to nodes that are located within the coverage zone of the sender. Each node receives this RREQM, adds its address to the “Route” field, and will forward it to other nodes if they are located in the request zone. However, to those nodes that receive the RREQM before (i.e., same packet ID) or are located outside the request zone, they will discard the RREQM. Afterward, the RREQM moves through intermediate nodes until reaches the *D* node. As soon as the *D* node acquires several RREQMs from different nodes, it generates a RREPM for every RREQM. Figure 7 shows the information of the RREPM.

The nodes that exist in the coverage area of the *D* node will receive the RREPM. If they find their address in the “Route” field, they will update the content of “Route temperature” and “Route busyness” by adding their information and forwarding the RREPM. Then, the RREPM will move through the intermediate nodes until reaching the *S* node. It can be observed that the earlier process may take some time (i.e., not immediately). Consequently, there is a period of time named the Time Out Period (TOP). In other words, the *D* node must wait and then make a decision for the next process.

**Figure 8:**
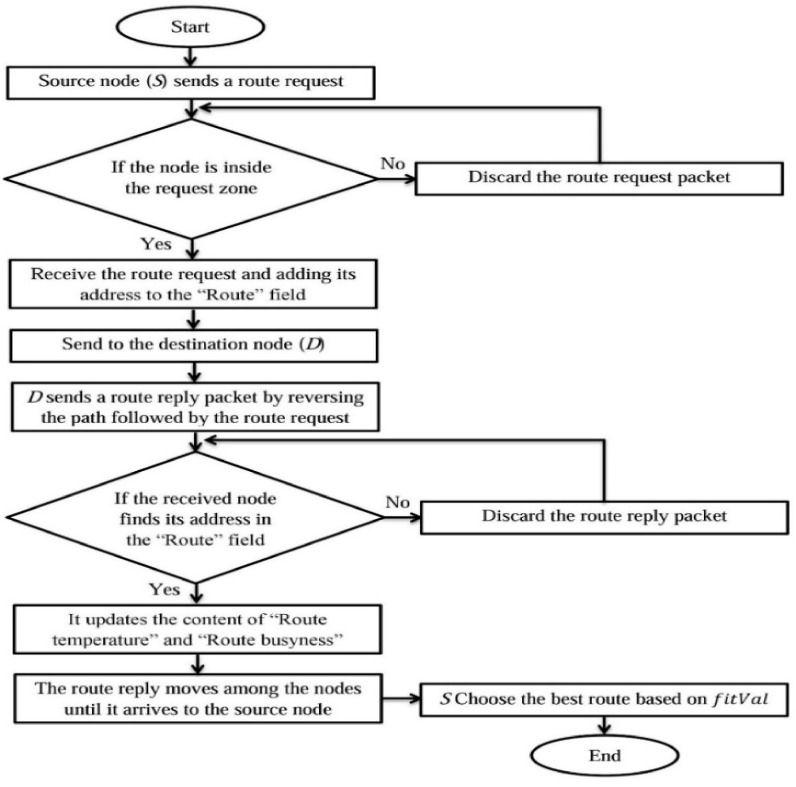
Flowchart of LARRR protocol.

However, in the case where no routes exist to the *D* node after the TOP or the *S* node does not receive the RREPM, the *S* node will receive a route error message, and it creates a new route discovery process to search for other routes to send another data packet to the *D* node. Thus, the data packet of the broken path should be removed if the TOP is elapsed. In other words, the RREQM, RREPM, and data packet should all arrive through the TOP. Consequently, the node should select the best route in order to obtain a better performance of the network with respect to receiving as many packets as possible, reduce the routing overhead, reduce the delay, and reduce the energy consumption. In the LARRR protocol, routes that include nodes with high temperatures should be chosen because these nodes will fail shortly due to the fire. Therefore, consuming the energy of these nodes before they totally fail is considered a cost-efficient method for the purpose of saving other nodes’ energy and then increasing the network’s lifetime. However, if a node with high temperature is constantly selected, the route of that node will be very congested. Accordingly, the proposed LARRR protocol is based on three criteria for obtaining a fitness value to select the best route, where each route is set with a fitness value to evaluate the quality of routes and then to determine the best route. The routes in the LARRR protocol consider the route length, node’s temperature, and the packets number in the node’s buffer. The fitness value of the LARRR protocol can be obtained as follows:

(1) $$fitVal=RL+\left(1-\mathrm{Max}\left(Temp\right)\right)+Max\left(B\right)$$

where *RL* (*Route Length*) indicates the hops number from the *S* to the *D*, *Max (Temp)* indicates the highest temperature of a node (i.e., 130 °C), while *Max (B)* indicates the maximum number of existing packets within node buffers. It is worth mentioning that a node should select the best route based on obtaining the lowest fitness value. Furthermore, the transmission of a RREQM should be stopped after a period of time for the purpose of reducing the routing overhead of the proposed LARRR protocol. Based on the tuning of our simulation, the best value of this period is selected at 1.5 s, where the overhead will increase in the network if this period is selected more than 1.5. Otherwise, there will be not enough route requests in the network if this period is selected less than 1.5. However, nodes used in this simulation are usually provided with restricted resources. Thus, it is not required to keep all offered routes, where nodes will keep the best route only through the TOP. The highest temperature a node can tolerate is 130 °C, where the node will totally burn and die at higher temperatures. The flowchart of the LARRR protocol is depicted in Figure 8.

**Figure 9:**
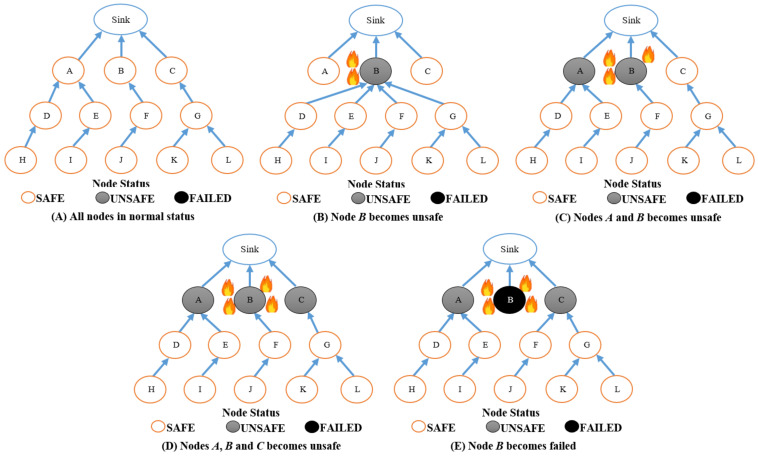
The diagram of the proposed LARRR protocol.

Furthermore, Figure 9 displays the diagram of the LARRR protocol for selecting the best route with respect to the fitness value. According to Figure 9B, the *B* node becomes unsafe. In other words, it senses a high temperature. Thus, all neighbors of the *B* node (i.e., *D*, *E*, *F*, and *G* nodes) change their parent to the *B* node in order to use and exploit the energy of the *B* node and save the power of the other nodes. Hence, the network lifetime will increase. In Figure 9C, the temperature of the *A* node is also increased. Therefore, the parent of nodes *D* and *E* is changed to the *A* node. Meanwhile, the buffer of node *B* is nearly filled by the data. Thus, the parent of the *G* node will change to the *C* node in order to avoid dropping packets from the *B* node. Figure 9D shows that *A*, *B*, and *C* nodes all became unsafe, while Figure 9E shows that the *B* node is already failed and burned because its temperature reaches 130 °C. In this condition, the *F* node has to select either the *A* node or the *C* node based on temperature and the node’s buffer. Thus, the *F* node selects the *A* node because the temperature and buffer of the *A* node are higher than those of the *C* node. Consequently, the proposed LARRR protocol takes into account the nodes, which sense high temperatures and also the buffer of nodes. This leads to avoiding the bottleneck problem occurring and dropping many data packets.

## 6. Simulation Setup

The PCE-OLSR protocol is chosen to compare with the proposed LARRR protocol. To adopt these routing protocols in the fire conditions, the simulation environment is deemed to be a square forest (i.e., 1000 × 1000 m^2^). In addition, 50 nodes are deployed in order to scan the forest. Table 1 depicts the simulation parameters for the proposed LARRR protocol.

**Figure 10:**
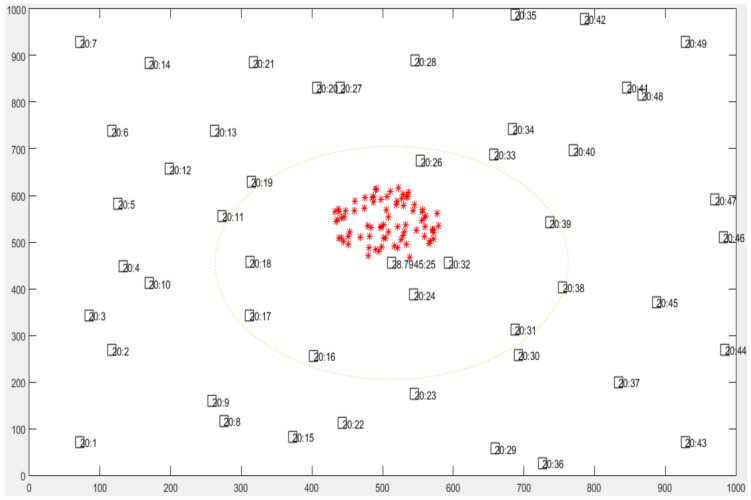
Simulation model of forest fire detection.

The nodes in this simulation have a coverage area with a radius of 250 m. In addition, each node moves in a circle form, where its diameter is up to 125 m. The initial energy of each node is equal to 2 kJ [36]. In normal conditions, the node’s temperature is 20 °C. When the node scans a fire area, its temperature increases linearly with a step equal to 0.0055 °C. The experiment time of this simulation is 1500 s, and the forest fire will be initiated after 400 s of experiment time. The fire’s location is selected in the center of the forest, as shown in Figure 10.

The fire is assumed to be a circle, increasing with a speed of 0.5 m/s. In addition, the largest radius of the fire is 300 m. The packets inside the node are generated by using 2 Poisson variables. The first variable is used for generating the time with a mean equal to 15 s. Meanwhile, the second variable is used for generating the packets with a mean equal to 1 packet. The lifetime of each packet is 15 s. The simulation is conducted using MATLAB R2017B as the simulator software on a PC (Windows 10) with 6 GB RAM and a 1 TB HDD.

## 7. Evaluation Measurements

There are many measurements that can be used to evaluate the network performance. The researchers in [37] and [38] presented various evolution measurements such as the percentage of acquired packets and the delay. In spite of these, a simulation is required with regard to the node mobility pattern, as stated by [39] who proposed that an arbitrary waypoint mobility model can be used to ascertain the delay. Furthermore, we use different evaluation measurements such as End-to-End Delay (E2E delay), Packet Delivery Ratio (PDR), Energy Consumption (EC), and Routing Overhead (RO). These measurements are explained and calculated in the following subsections.

### 7.1. PDR

This is defined as the percentage of the number of received packets through the *D* node to the number of transmitted packets through the *S* node [40]. Furthermore, the calculation of PDR can be performed as shown in Equation (2).

(2) $$\mathrm{PDR}\;\left(\%\right)=\frac{number\;of\;packets\;received}{number\;of\;packets\;sent\;\;\;}\times 100$$

### 7.2. E2E Delay

This indicates the time of packets that are sent successfully from the *S* node to the *D* node within the network [41]. In addition, the calculation of E2E delay can be performed as shown in Equation (3), where n refers to the total number of packets that are sent successfully and p refers to the packet number.

(3) $$E2E\;\mathrm{Delay}\;\left(s\right)=\frac{\sum_{p=1}^{n}(arrive\;time_{p}-send\;time_{p})}{number\;of\;packets\;}$$

### 7.3. RO

The RO indicates the entire number of routing packets that are produced by a routing protocol within the network [42]. Furthermore, the calculation of RO can be performed as shown in Equation (4).

(4) $$\mathrm{RO}=\frac{number\;of\;routing\;packets\;}{number\;of\;routing\;packets+number\;of\;data\;packets\;sent\;\;\;}$$

### 7.4. EC

This indicates the total consumed energy by all nodes within the network, where this measurement computes the network’s lifetime [42]. In addition, the calculation of EC can be performed as shown in Equation (5).

(5) $$\mathrm{EC}=\frac{sum\;of\;energy\;consumed\;by\;all\;nodes}{network\;initial\;energy\;}$$

## 8. Simulation Results and Discussion

**Figure 11:**
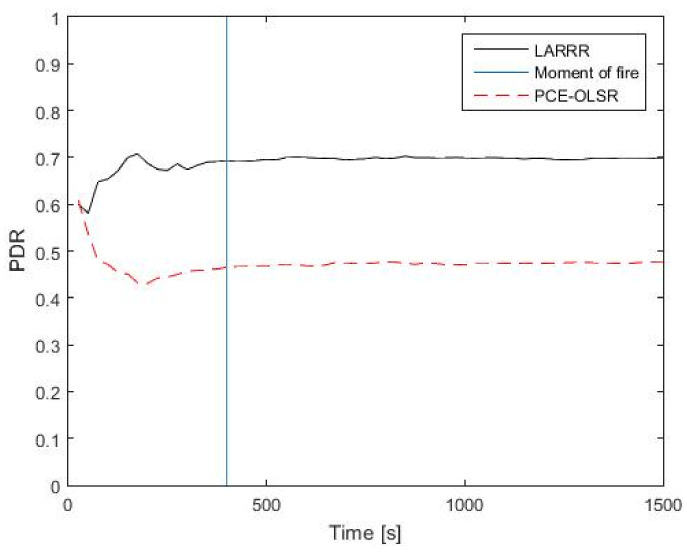
PDR results between LARRR and PCE-OLSR.

The performance of the LARRR protocol is assessed and compared with a recently developed protocol to identify which of these protocols (i.e., LARRR or PCE-OLSR) is more reliable and efficient in the detection of a forest fire. The evaluation and comparison between these routing protocols are conducted using PDR, EC, E2E delay, and RO. Figure 11 shows the comparison between the LARRR protocol and PCE-OLSR protocol with respect to the PDR. Due to the fire conditions, the routing protocols show different performances before and after the fire ignition. Before the fire ignition, the proposed LARRR protocol achieves a 69.11% PDR value, while the PCE-OLSR protocol achieves a 46.17% PDR value. After the fire ignition, the LARRR protocol achieves a 70% PDR value and the PCE-OLSR protocol achieves a 47.74% PDR value.

Thus, the LARRR protocol outperforms the PCE-OLSR in the PDR, where the LARRR protocol achieves a higher amount of received data as compared to the PCE-OLSR. Therefore, the LARRR achieves a better PDR performance of 46.6% as compared with the PCE-OLSR. This is interpreted by the fact that PCE-OLSR selects the node with the highest temperature only to route the messages toward it. Consequently, this leads to the bottleneck problem caused by the high busyness of the node’s buffer. In the proposed LARRR protocol, the buffer busyness is considered for routing the messages along with the temperature.

**Figure 12:**
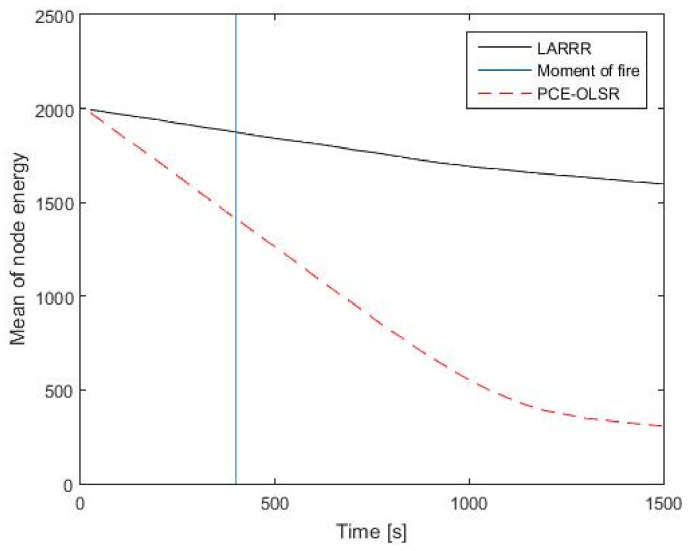
EC results between LARRR and PCE-OLSR.

In addition, Figure 12 presents the comparison results between LARRR and PCE-OLSR in the energy consumption. Before the fire ignition, the average energy of the LARRR protocol is equal to 1883 joules and the average energy of the PCE-OLSR proto-col is equal to 1450 joules. Furthermore, the average energies of LARRR and PCE-OLSR protocols after the fire ignition are 1597 joules and 308 joules, respectively. It is obvious that the node energy in the proposed LARRR is larger than that of PCE-OLSR. Hence, the LARRR protocol outperforms PCE-OLSR with 418.5% in the reduction in the consumed energy. The PCE-OLSR method is based on the OLSR protocol, which is regarded as a type of proactive protocol. Consequently, it consumes more energy due to the control messages that are exchanged in order to maintain the route availability. In the LARRR protocol, the routes are generated only when they are needed.

**Figure 13:**
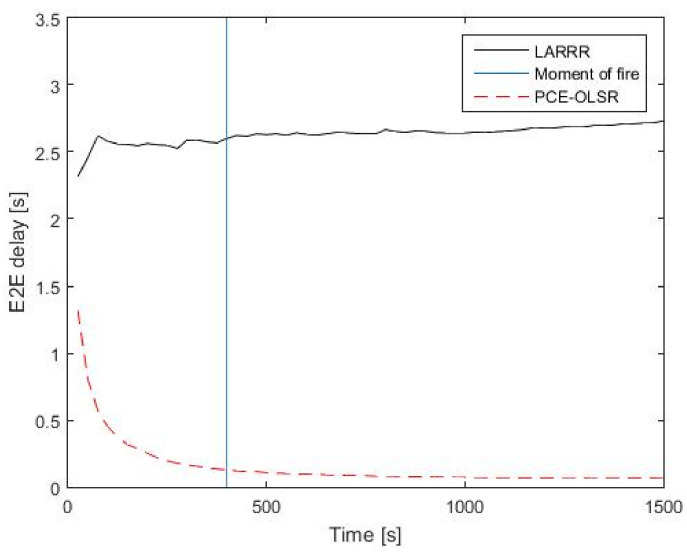
E2E delay results between LARRR and PCE-OLSR.

Figure 13 shows the performance evaluation of LARRR and PCE-OLSR protocols in the E2E delay. Before the fire ignition, the performance of LARRR achieves a 2.568 s average E2E delay value and the PCE-OLSR protocol obtains a 0.1353 s average E2E delay value. After the fire ignition, the LARRR achieves a 2.733 s average E2E delay value, while the PCE-OLSR achieves a 0.0701 s average E2E delay value. It is obvious that the value of E2E delay in the LARRR protocol increases, while the value of E2E delay in PCE-OLSR decreases. This is because nodes in the PCE-OLSR protocol generate the routes even before they are required, while the routes in the LARRR protocol will be generated only when they are required. Therefore, the process of route discovery in the PCE-OLSR is executed faster than those that exist in the LARRR protocol.

**Figure 14:**
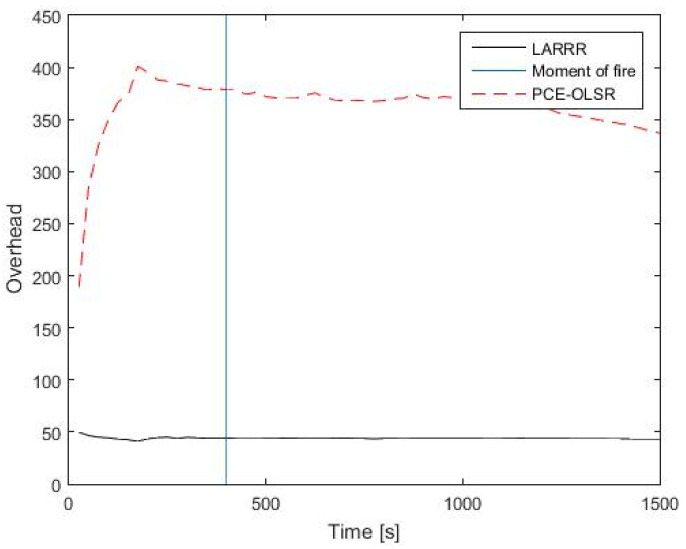
RO results between LARRR and PCE-OLSR.

Furthermore, the results of RO for LARRR and PCE-OLSR protocols are shown in Figure 14. The RO of the PCE-OLSR protocol is much higher than the RO of the proposed LARRR protocol, where the LARRR protocol achieves a 43.8 overhead value before the fire ignition. After the fire ignition, the LARRR protocol achieves a 43.04 RO value. Meanwhile, the RO results of the PCE-OLSR protocol before and after the fire ignition are 379.5 and 336.8, respectively. Therefore, the RO is enhanced in the proposed LARRR protocol compared to PCE-OLSR by 87.22%.

This is because nodes in the PCE-OLSR have a table of routing information in order to establish the routes, and these nodes update their routing information periodically, which leads to an increase in the overhead in the overall network, while the proposed LARRR protocol is considered as a type of reactive routing protocol, wherein the nodes in this protocol create routes when they are needed only. For instance, when a node needs to transmit a packet, a process called the Route Discovery Process (RDP) will start to search and find a route to the *D* node. This RDP is finished once a route is discovered or all potential routes to the *D* node have been examined. In addition, Table 2 shows the comparison between the proposed LARRR protocol and the PCE-OLSR protocol in terms of the methodology of each protocol in the detection of forest fires.

## 9. Conclusions

In this paper, we present a new protocol called LAR-Based Reliable Routing (LARRR) in the detection of wildfire. The proposed LARRR protocol is performed using three criteria: route length, temperature sensing, and route busyness (i.e., the number of packets within nodes buffers). The proposed LARRR protocol is assessed and compared with the PCE-OLSR protocol with respect to E2E Delay, PDR, EC, and RO. The simulation outcomes show that the proposed LARRR protocol outperforms the PCE-OLSR in terms of a PDR of 46.6%, energy consumption of 418.5%, and RO of 87.22%. Meanwhile, the PCE-OLSR protocol outperforms the LARRR protocol with respect to E2E delay. This is because the PCE-OLSR is based on the proactive protocol, where the nodes establish the routes before they are required. Meanwhile, the LARRR protocol is based on the reactive protocol, where the nodes establish the routes only when they are needed. Therefore, the process of route discovery in the PCE-OLSR protocol is implemented faster than the process existing in the LARRR protocol. Future work can include using the development process of the LARRR protocol in another routing protocol such as OLSR for the purpose of elevating its performance in forest fire detection.

## Figures and Tables

**Table 1:** Simulation parameters.

Parameters	Values
Experiment time	1500 (s)
Average of logging data	25 (s)
The ignition time of fire	400 (s)
Nodes number	50 (node)
Nodes energy	2 (kJ)
Coverage area radius	250 (m)
Average size of packet	80 (bite)
Normal node temperature	20 (degree)
Node speed	(7.5–12.5) m/s
Fire speed	0.5 m/s
Fire radius	300 (m)
Environment size	1000 × 1000 (m^2^)
Start point of fire	The forest center
Buffer size of data	100 (packet)
Lifetime of data packet	15 (s)
Interval arrival time	15 (s)
Generation mean of data packet	1 (packet)
Timeout of RREQM	1.5 (s)
Buffer size of RREQM	5000 (packet)
Buffer size of RREPM	1000 (packet)
Temperature increment	0.0055 degree/10 ms
Time unit	10 ms
Simulator	MATLAB

**Table 2:** Comparison of the methodology between the proposed LARRR and PCE-OLSR.

LARRR Protocol	PCE-OLSR Protocol
In the proposed LARRR protocol, the routing is performed based on three criteria: the route length between nodes, the temperature sensing, and the route busyness (i.e., node’s buffer).	In the PCE-OLSR protocol, the only criteria are the number of hops and the temperature.
2. The category of LARRR is an on-demand routing protocol.	2. The PCE-OLSR protocol is based on a proactive routing protocol.
3. The LARRR uses the location information of sensor nodes.	3. The PCE-OLSR protocol ignores the location information of sensor nodes.
4. The nature of the criteria in LARRR is continuous.	4. The nature of the criterion in PCE-OLSR is binary.

## Data Availability

Not applicable.
